# The PD Effluentome—A Multi-Omics Atlas Defining the Composition, Transport Dynamics, and Molecular Origin of Peritoneal Dialysis Effluent

**DOI:** 10.3390/medsci14040496

**Published:** 2026-08-19

**Authors:** Rebecca Herzog, Fabian Eibensteiner, Florian M. Wiesenhofer, Lisa Daniel-Fischer, Anja Wagner, Markus Unterwurzacher, Isabel J. Sobieszek, Juan Manuel Sacnun, Michael Böhm, Andreas Vychytil, Christoph Aufricht, Klaus Kratochwill

**Affiliations:** 1 Division of Pediatric Nephrology and Gastroenterology, Department of Pediatrics and Adolescent Medicine, Comprehensive Center for Pediatrics, Medical University of Vienna, 1090 Vienna, Austria; fabian.eibensteiner@meduniwien.ac.at (F.E.); lisa.daniel-fischer@meduniwien.ac.at (L.D.-F.); anja.wagner@meduniwien.ac.at (A.W.); markus.unterwurzacher@meduniwien.ac.at (M.U.); isabel.sobieszek@meduniwien.ac.at (I.J.S.); juan.sacnun@meduniwien.ac.at (J.M.S.); michael.boehm@meduniwien.ac.at (M.B.); christoph.aufricht@meduniwien.ac.at (C.A.); 2 Christian Doppler Laboratory for Peritoneal Regulation of the Immune-Metabolic Ecosystem in Peritoneal Dialysis (CDL-PRIME-PD), Department of Pediatrics and Adolescent Medicine, Medical University of Vienna, 1090 Vienna, Austria; 3 Core Facility Proteomics, Medical University of Vienna, 1090 Vienna, Austria; 4 Department of Medicine III, Division of Nephrology and Dialysis, Medical University of Vienna, 1090 Vienna, Austria; andreas.vychytil@meduniwien.ac.at

**Keywords:** liquid biopsy, multi-omics, peritoneal solute transfer, peritoneal membrane, peritoneal equilibration test

## Abstract

**Background**: Peritoneal dialysis (PD) effluent of kidney failure patients represents an accessible liquid biopsy of the peritoneal cavity, yet the mechanisms determining its molecular composition remain poorly understood. We applied an integrative multi-omics approach to characterize the composition, transport dynamics, and molecular origin of the PD effluentome. **Methods**: Cell-free effluent, effluent cells, and plasma were collected from stable PD patients during standardized peritoneal equilibration tests in a randomized clinical trial. Targeted metabolomics, proteomics, and transcriptomic profiling were integrated with a reference human plasma proteome to investigate temporal molecular changes, peritoneal transport characteristics, and protein origin. **Results**: A total of 207 metabolites and 2970 proteins were identified in PD effluent. Metabolites exhibited distinct class-specific transport kinetics, with rapid equilibration of amino acids and biogenic amines, whereas lipids remained markedly underrepresented despite prolonged dwell times, indicating that transport is governed by physicochemical properties beyond molecular size alone. The effluent proteome underwent concordant alteration, with dwell time-dependent enrichment of pathways related to extracellular matrix organization, angiogenesis, coagulation, and tissue repair. Integrative analysis of the effluent proteome, effluent-cell transcriptome, and human plasma proteome resolved distinct plasma-associated, effluent cell-associated, resident peritoneal tissue-associated, and mixed-origin protein populations. **Conclusions**: This study establishes the first systems-level approach describing the composition, transport dynamics, and molecular origin of the PD effluentome. By transforming PD effluent into a biologically interpretable molecular readout of peritoneal membrane biology, this work provides a reference for the mechanistic interpretation of effluent-derived biomarkers and supports future therapeutic monitoring and precision medicine in PD.

## 1. Introduction

Peritoneal dialysis (PD) is the leading home-based kidney replacement therapy, generating liters of peritoneal dialysis effluent (PDE) during each treatment. Although routinely discarded as waste, PDE represents an abundant and readily accessible liquid biopsy with considerable biomarker potential. PD provides greater treatment flexibility and quality of life than conventional in-center hemodialysis and is the most common modality for treating children with kidney failure [1,2,3]. During PD, hyperosmolar dialysis fluid is instilled into the peritoneal cavity, where the peritoneal membrane serves as a semipermeable barrier for the exchange of water and solutes between blood and dialysate. Over the course of a treatment dwell, excess fluid, metabolic waste products, including uremic toxins, plasma proteins, and locally released molecules accumulate in the dialysate, which is subsequently drained as PDE [4]. PDE is one of the few biofluids that can be sampled repeatedly and without additional burden to the patient throughout chronic kidney replacement therapy. As such, it represents a unique liquid biopsy of the peritoneal microenvironment with considerable, yet largely untapped, potential for biomarker discovery and mechanistic investigation.

Beyond its diagnostic value, accumulating evidence indicates that PDE reflects a biologically active peritoneal microenvironment that influences peritoneal cell function rather than representing a passive ultrafiltrate of plasma [5,6,7,8]. Collectively, the molecules present in PDE, including proteins, metabolites, nucleic acids, and cells constitute what we define here as the PD effluentome; a dynamic molecular ecosystem shaped by both systemic physiology and local cellular activity. Moreover, its composition evolves continuously throughout a dwell as systemic transport, local secretion, cellular turnover, and metabolic activity collectively determine the molecular composition of the effluent.

The current concept of peritoneal transport is largely based on the classical three-pore model, which describes water and solute movement through ultrasmall aquaporins, small intercellular pores, and large pores within the peritoneal vasculature [9,10,11]. This framework explains the physical principles of ultrafiltration and diffusion but does not account for molecule-specific properties such as charge, hydrophobicity, protein function and binding, molecular size, or local cellular secretion, that may influence the transport and accumulation of proteins and metabolites in PDE [12,13]. Interpretation of the effluent is further complicated by chronic exposure to dialysis fluid, which induces inflammation, oxidative stress, angiogenesis, fibrosis, and progressive remodeling of the peritoneal membrane [14]. Together, these processes generate a dynamic mixture of plasma-derived and locally produced molecules whose origin and temporal dynamics remain incompletely understood.

Neither current transport models nor isolated biomarker approaches fully capture the molecular complexity of PDE. Consequently, an integrative multi-omics approach may be attractive to characterize the PD effluentome, disentangle the contributions of systemic filtration and local production, and define the temporal dynamics that shape its molecular composition during a PD dwell.

Here, we establish an integrative, time-resolved multi-omics framework to comprehensively characterize the PD effluentome using samples collected during a prospective randomized clinical trial. By combining proteomics, metabolomics, and transcriptomics we define the molecular composition of the PD effluentome, thereby providing a molecular framework for mechanistic studies and future biomarker discovery.

## 2. Materials and Methods

Patient cohort and randomized clinical trial: Samples were collected of patients included in a pilot randomized clinical trial with a cross-over design. The study was approved by the local ethics committee of the Medical University of Vienna (EK1755/2012 and EK 2035/2015), registered (EudraCT 2012-004004-36), and was carried out in accord with the Declaration of Helsinki. All patients gave written informed consent prior to enrolment. Details of the study protocol, inclusion criteria, patient details, clinical and safety parameters, as well as the primary and explorative outcomes have been previously described [15]. In brief, six clinically stable PD patients (4 male, 2 female) aged 52.5 (45–68) years, treated with PD for 17.8 (4.9–48.1) months were included. Two PD exchanges were performed with the respective test fluids, with a 16 h overnight dwell immediately followed by a 4-h standard peritoneal equilibration test (PET) [16,17]. For the overnight dwell, patients were treated with a glucose-based PD fluid according to their prescription. For the 4 h PET dwell, patients were treated with glucose-based PD fluid (Physioneal 40 with 3.86% glucose, Baxter, Castlebar, Ireland) or the same PD fluid supplemented with 8 mM l-alanyl-l-glutamine (Dipeptiven 200 mg AlaGln/mL, Fresenius-Kabi Healthcare, Bad Homburg, Germany (not used in this study)). Study visits were separated by a wash-out period (≥ 28 days). PDE samples were taken at the end of the overnight dwell and after 0, 1 and 4 h of the subsequent PET. Plasma samples were taken at the 2 h time point of the PET. Plasma samples from 6 healthy donors (3 male, 3 female) were taken after informed consent.

PDE and plasma sample preparation: PDE was collected in standard 9 mL native collection tubes (Vacuette, Bio-Greiner-One, Kremsmünster, Austria) at the 0 and 1 h time points and centrifuged (250× *g*, 10 min), aliquots of cell-free supernatant were stored at −80 °C until further analyses. From the overnight dwell and the 4-h PET was completely drained and collected in the respective drain bags. The total effluent volume was centrifuged in 650 mL aliquots (250× *g*, 30 min) aliquots of cell-free supernatant were stored at −80 °C until further analyses. 1 L of cell-free PDE was immediately processed using combinatorial peptide ligand libraries (CPLL) for proteomic analysis as described below. Cell pellets obtained from the 4-h PET were used for transcriptomic analyses as previously described [15]. Peripheral blood was collected into ethylenediaminetetraacetic acid (EDTA) tubes, centrifuged (3000 rpm, 10 min), and plasma aliquots were stored at −80 °C until further analyses.

Targeted metabolomics: Metabolites in cell-free PDE (30 µL) and plasma (10 µL) samples were analyzed using the AbsoluteIDQ p400 HR kit (Biocrates Life Sciences AG, Innsbruck, Austria) according to the manufacturers’ instructions. Samples were analyzed by MS (Orbitrap Q Exactive (Thermo Fisher Scientific, Waltham, MA, USA)) run in multiple reaction monitoring (MRM) mode for both liquid chromatography (LC, Vanquish ultra-high-performance LC system (Thermo Scientific, Waltham, MA, USA)) and flow injection analysis (FIA) analysis. Data processing and validation were in performed using the MetIDQ-6.4.8 software (Biocrates Life Sciences AG, Innsbruck, Austria) per manufacturers’ instructions. Molecular masses and solubility of metabolites were obtained from the Human Metabolome Database (www.hmdb.ca, accessed on 20 June 2026). Six metabolites (Asp, Ac-Orn, Putrescine, Spermine, Spermidine, alpha-AAA) were excluded during raw data processing and quality control due to analytical inconsistencies or no detectability in all samples.

Proteomics analysis: Cell-free PDE obtained after the overnight dwell and the 4-h PET was subjected to depletion of high-abundance proteins and enrichment of low-abundance proteins using a previously established CPLL protocol (ProteoMiner Beads, Bio-Rad, Hercules, CA, USA) with minor modifications [18]. 1 L of cell-free PDE was incubated with 250 μL ProteoMiner bead suspension (corresponding to a 50 μL settled bead volume) on a roller mixer overnight at 4 °C. The beads were allowed to sediment for 30 min, the supernatant was carefully aspirated and the beads were recovered and centrifuged (100× *g*, 10 min). Beads were recovered in 1 mL supernatant and transferred to Mini Bio-Spin chromatography columns (Bio-Rad). Following four washing steps with washing buffer (5 min, 150 μL, 150 mm NaCl, 10 mm NaH_2_PO_4_, pH 7.4) and once with 200 μL deionized water, proteins were eluted by four sequential elutions (15 min, 50 μL, 8 m urea, 2% 3-[(3-cholamidopropyl) dimethylammonio]-1-propanesulfonate (CHAPS) in 5% acetic acid). Protein samples were precipitated (100% acetone, overnight, −20 °C), washed with ethanol and resuspended in 150 μL buffer (30 mm Tris-HCl, pH 8.5, 7 M urea, 2 M thiourea, 4% CHAPS, 1 mM EDTA, 1 tablet of Complete Protease Inhibitor (Roche, Basel; Switzerland) and 1 tablet of phosphatase inhibitor (PhosSTOP, Roche, Basel; Switzerland) per 100 mL. Protein concentration was determined using the 2D Quant kit (GE Healthcare, Uppsala, Sweden) per the manufacturer’s instructions. Filter-Aided Sample Preparation (FASP) was performed using a 30 kDa molecular weight cut-off filter (VIVACON 500; Sartorius Stedim Biotech, Goettingen, Germany). 100 μg of each sample was mixed with 180 μL UA buffer (8 M urea in 100 mM Tris-HCl, pH 8.5) in the filter unit and centrifuged (14,000× *g*, 15 min, 20 °C). Following a second washing step with UA (200 μL), the proteins were alkylated (100 μL 50 mM iodoacetamide, 30 min, RT). Following 3 UA washes (100 μL) and 3 more washes with triethylammonium bicarbonate buffer (TEAB) buffer (50 mM, 100 μL), proteins were digested with trypsin (overnight, 37 °C). Peptides were recovered using 40 μL 50 mM TEAB buffer, followed by 50 μL 0.5 M NaCl. Acidified tryptic peptides were concentrated and desalted using C18 spin columns (The Nest Group, Southborough, MA, USA).

Tandem mass tag (TMT) derivatization: Following enzymatic digestion, proteins were labeled with isobaric tags (TMT 11-plex, Thermo Fisher Scientific, Waltham, MA, USA). TMT labelling was performed according to the instructions provided by the manufacturer. Pooled samples were concentrated and desalted with C18 macrospin columns (30–300 μg, The Nest Group). Eluates were lyophilized in a vacuum concentrator and reconstituted in 20 mM ammonia formate buffer (pH 10) before fractionation at basic pH.

2D-RP/RP liquid chromatography-mass spectrometry for proteomics: Two-dimensional reversed-phase (RP) liquid chromatography was performed by reverse-phase chromatography at high and low pH. In the first dimension, peptides were separated on a Gemini-NX C18 (150 × 2 mm, 3 μm, 110 Å, Phenomenex, Torrance, CA, USA) in 20 mM ammonia formate buffer (pH 10) and eluted over 45 min by a 5–70% acetonitrile gradient at 100 μL/min using an Agilent 1200 high performance liquid chromatography (HPLC) system (Agilent Biotechnologies, Palo Alto, CA, USA). 96 time-based fractions were collected and pooled into 40 HPLC vials based on the UV-trace at 214 nm. Samples were acidified by the addition of 5 μL of 5% formic acid, the organic solvent was removed in a vacuum concentrator, and samples were reconstituted in 5% formic acid. Every fraction was injected once. Mass spectrometry was performed on an Orbitrap Fusion Lumos Tribrid mass spectrometer (Thermo Fisher Scientific) using the Xcalibur version 2.1.0 coupled to an Agilent 1200 HPLC nanoflow system (dual pump system with one trap-column and one analytical column) via a nano-electrospray ion source using a liquid junction (Proxeon, Odense, Denmark). Analyses were performed in a data-dependent acquisition mode using a top-15 higher-energy collision-induced dissociation (HCD at 35) method for peptide identification plus relative quantitation of TMT reporter ions. Dynamic exclusion for selected ions was 30 s. A single lock mass at *m*/*z* 445.120024 was employed. The maximal ion accumulation time allowed for MS mode in the Orbitrap was 50 ms, and the accumulation time for HCD was 86 ms. Automatic gain control (AGC) was used to prevent overfilling of the ion traps with AGC targets and set to 2 × 10^5^ and 10^5^ ions for MS1 and MS2, respectively. A resolution of 120,000 and 50,000 (at *m*/*z* 400) was chosen for MS1 and MS2, respectively. The threshold for triggering MS2 scans was set to 20,000 counts.

LC-MS data analysis for proteomics: The acquired raw MS data files were processed with Proteome Discoverer (v 2.2, Thermo Scientific). The precursor window was set to 350—5000 Da. 2 missed cleavages were allowed and the minimum peptide length was set to 6. Precursor mass tolerance was 10 ppm, fragment mass tolerance 0.025 Da. Oxidation, deamidation and terminal acetylation were set as dynamic modifications. Static modifications were TMT and carbamidomethylation. FDR (0.01 threshold) was controlled at the peptide and protein level. Proteins were grouped according to the strict parsimony principle. Two-step normalization of the protein expression matrix was done: (i) protein loadings were adjusted on the peptide level by scaling the peptide sum abundance to the highest channel, and (ii) protein abundances were scaled to an internal standard contained in every TMT-batch to ensure the stability of the data across multiple batches. The internal standard was a mixture of all samples included in the experiment, pooled in equal amounts (13 µg per sample). Another standard, termed “global standard” was included to enable cross-experiment normalization to future studies. It consists of 208 PDE samples in our bio-bank. Only unique peptides were quantitated. Only peptides and proteins with FDR <0.01 are reported, and single peptide IDs were excluded from the analysis.

Transcriptomics analysis of effluent cells: RNA sequencing of effluent cells obtained from the 4-h PET was performed and published previously [15]. For the present study, the original sequencing data were reprocessed for integrative multi-omics analysis.

Publicly available reference datasets: Protein abundance in healthy human plasma was obtained from the Human Plasma Proteome Project (HPPP) Database (PeptideAtlas, April 2020 release) [19]. In silico predictions of protein secretory signals were obtained from the Human Protein Atlas [20].

Bioinformatics and statistical analysis: All statistical analyses were performed in R (version 4.5.1) [21]. Unless otherwise specified, analyses included all quantified measurements, including values below the limit of detection (LOD), to preserve information on metabolite appearance during dwell progression. Molecular masses and aqueous solubility of metabolites were obtained from the Human Metabolome Database (www.hmdb.ca).

Metabolomics data analyses: To investigate metabolite equilibration during the PET, the initial time point immediately after dialysate instillation (0 h) was excluded from statistical testing because residual intraperitoneal fluid resulted in increased variability. Dwell time-dependent changes were assessed for each metabolite using repeated-measures ANOVA followed by Tukey’s post hoc test. To visualize equilibration kinetics across metabolite classes, median concentrations of significantly regulated metabolites were calculated for each time point (including 0 h), z-score transformed, and normalized to the lowest scaled median concentration within each metabolite class. Hierarchical clustering of individual metabolites was performed using z-score normalized concentrations. Peritoneal transport characteristics were assessed by calculating dialysate-to-plasma (D/P) ratios for metabolites detected above the LOD in at least 50% of samples (>3 observations) at each time point. Differences between plasma from PD patients and healthy controls were evaluated using Welch’s *t*-test.

Proteomics data analyses: Differential protein abundance between the 4-h PET and overnight dwell was assessed using the limma R-package) (v4.5.1) [22]. Proteins passing a significance threshold (false discovery rate adjusted *p*-value < 0.05 and log2 fold-change > 0.33) were subjected to downstream functional analyses. Functional enrichment analyses, including canonical pathways, diseases and biological functions, upstream regulator prediction, and interaction network analyses, were performed using Ingenuity Pathway Analysis (IPA; QIAGEN, Hilden, Germany). Protein interaction networks were generated using experimentally validated direct interactions from the Ingenuity Knowledge Base, and disconnected proteins were removed for visualization. Gene Ontology enrichment analysis was used to identify significantly enriched cellular compartments among dwell time-regulated proteins. To explore potential involvement of aryl hydrocarbon receptor (AHR) signaling, experimentally validated AHR target proteins were retrieved from the Ingenuity Knowledge Base. Associations between metabolite abundances and AHR-regulated proteins were evaluated using linear models including metabolite abundance and dwell time as explanatory variables.

Integrative multi-omics analyses: RNA sequencing data from effluent cells were reprocessed, transcripts with fewer than 10 sequencing reads were excluded prior to analysis. Protein abundances were estimated using TMT-corrected intensity-based absolute quantification (iBAQ), while transcript abundances were normalized as reads per kilobase per million mapped reads (RPKM) [23]. Protein and transcript identifiers were mapped using the UniProt ID Mapping service and manually curated where required. To determine the molecular origin of proteins detected in PDE, UniProt accessions were matched between the PDE proteome, effluent-cell transcriptome, and the Human Plasma Proteome reference dataset. Overlapping protein sets were subjected to Reactome pathway enrichment analysis, and parent pathway terms were extracted for visualization. Quantitative relationships between PDE proteins, plasma proteins, and cellular transcripts were assessed using rank-based correlations between matched protein iBAQ values, estimated plasma protein abundances, and transcript RPKM values.

## 3. Results

### 3.1. Time-Resolved Metabolomics Reveals Class-Specific Equilibration Kinetics During PD

**Figure 1:**
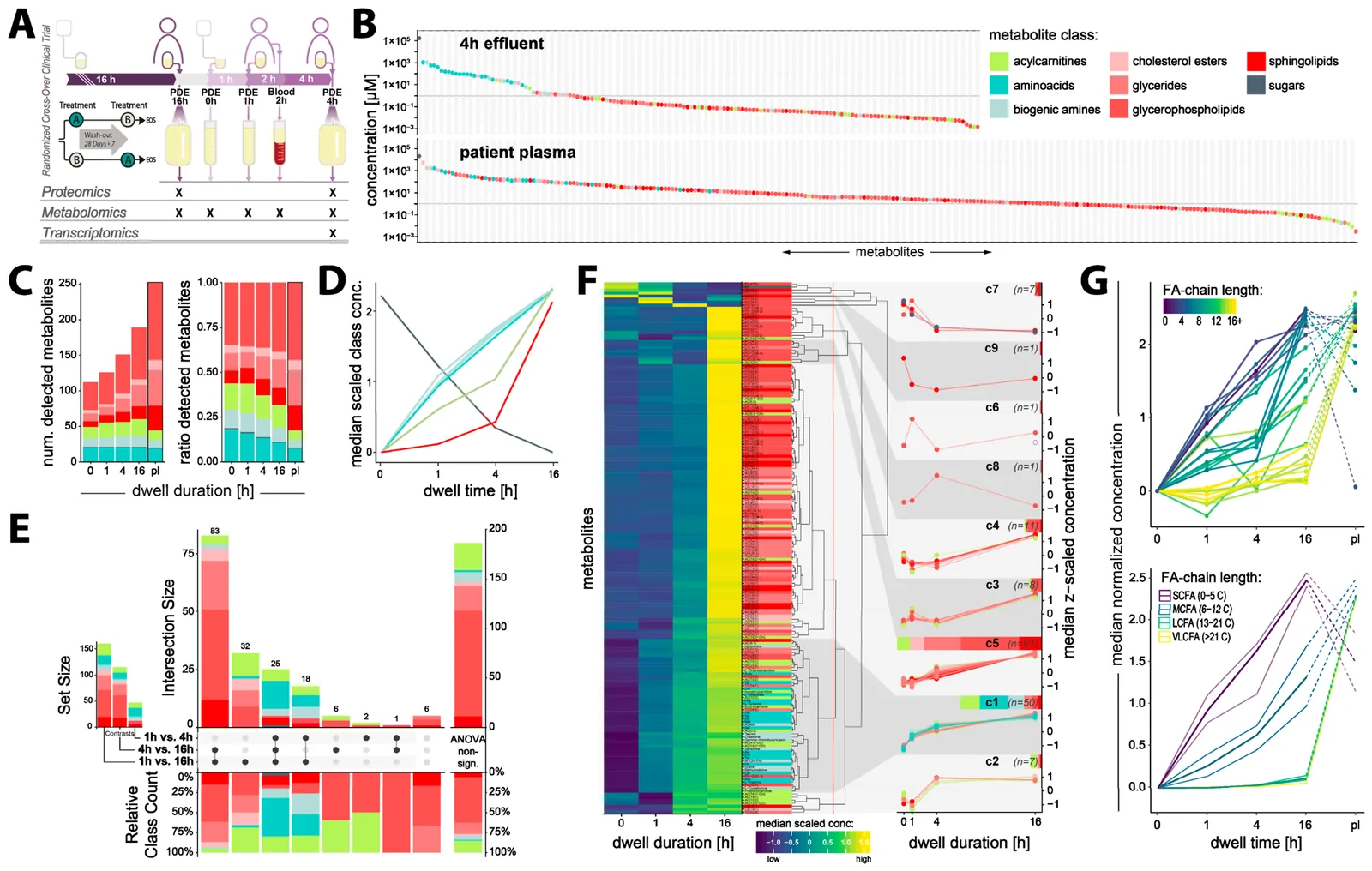
Time-resolved metabolomics profiling reveals dynamics of the peritoneal dialysis effluent metabolome during a standardized peritoneal equilibration test (PET). (**A**) Schematic overview of the study design. Cell-free PD effluent (PDE) was collected after the routine overnight dwell (16 h) and during a standardized 4-h PET (0, 1, and 4 h). Matched plasma samples were obtained at the 2-h PET time point. Samples were subjected to integrated multi-omics analyses. (**B**) Ranked abundance of metabolites in 4-h PDE and matched patient plasma. Amino acids and biogenic amines dominate the high-abundance range of PDE, whereas plasma exhibits a more even distribution across metabolite classes. Metabolites are only shown if they were detected in >50% of samples. Full version with metabolite labels Figure S1. (**C**) Number of quantifiable metabolites and metabolite class composition in PDE and plasma throughout the dwell. (**D**) Median scaled concentrations of metabolite classes during the PET and overnight dwell. Lipid classes are shown combined. Glucose (quantified as sum of hexoses, H1) showed a PD-specific dwell-time dependent decrease (gray line) (**E**) Significantly changing (ANOVA, Tukey’s post hoc) metabolites between sampling time points (pairwise comparisons). Absolute and relative distribution of significantly changing metabolites across metabolite classes are shown. (**F**) Hierarchical clustering of dwell time-dependent metabolites (scaled concentrations) identifies distinct concentration profiles during peritoneal equilibration. (**G**) Dwell time-dependent transport of acylcarnitines according to fatty acid chain length, demonstrating chain length-dependent equilibration kinetics. SCFA: short-chain fatty acids, MCFA: medium-chain fatty acids, LCFA: long-chain fatty acids, VLCFA: very long-chain fatty acids.

A total of 402 unique metabolites were targeted in PDE and plasma samples collected during the control phase of a prospective randomized crossover clinical trial (Figure 1A). PDE was sampled from the overnight dwell (16 h) and during a subsequent 4-h PET at 0, 1, and 4 h, with matched plasma obtained after 2 h. Overall, 207 unique metabolites were detected across all PDE samples and 247 metabolites in plasma (Table S1). Glucose (quantified as the sum of hexoses) was the most abundant metabolite in both compartments. Whereas the most abundant metabolites in PDE were predominantly amino acids (AAs) and biogenic amines (BAs), plasma exhibited a more even distribution of metabolite classes across the entire abundance range (Figure 1B and Figure S1). The number of detectable metabolites increased progressively throughout the dwell from 105 immediately after dialysate instillation to 142 after 4 h and 180 after the overnight dwell (Figure 1C), reflecting continuous solute accumulation within the peritoneal cavity.

We identified 175 metabolites whose concentrations changed significantly with dwell time. Most differences occurred between the 1 h and 16 h samples (160 metabolites) (Figure S2), indicating that molecular equilibration continued well beyond the standard PET. Temporal profiles differed markedly between metabolite classes: AAs and BAs exhibited rapid accumulation, whereas lipids accumulated more slowly and predominantly during the later stages of the dwell. Acylcarnitines displayed intermediate kinetics, consistent with progressive but non-linear equilibration (Figure 1D,E). To identify common transport and accumulation profiles, dwell time-dependent metabolites were subjected to in depth analysis. Hierarchical clustering supported the class-specific transport patterns, with 79% of metabolites grouping into two dominant clusters representing rapidly equilibrating hydrophilic metabolites (Cluster 1; 28.5%) and slowly accumulating predominantly lipophilic metabolites (Cluster 5; 50.8%) (Figure 1F). Short-chain acylcarnitines clustered with rapidly equilibrating hydrophilic metabolites (Cluster 1), whereas increasing fatty acid chain length (according increasing hydrophobicity), was associated with progressively delayed or absent equilibration, demonstrating the capability of this approach to measure the influence of physicochemical properties on transport (Figure 1G). Consistently, PDE accumulation decreased with increasing chain length and molecular mass (Figure S3), while hydroxylated and dicarboxylic acylcarnitines, which possess greater polarity and hydrophilicity, equilibrated more rapidly than their non-modified counterparts.

### 3.2. Selective Peritoneal Transport Shapes the PDE Metabolome During a Clinical PET

**Figure 2:**
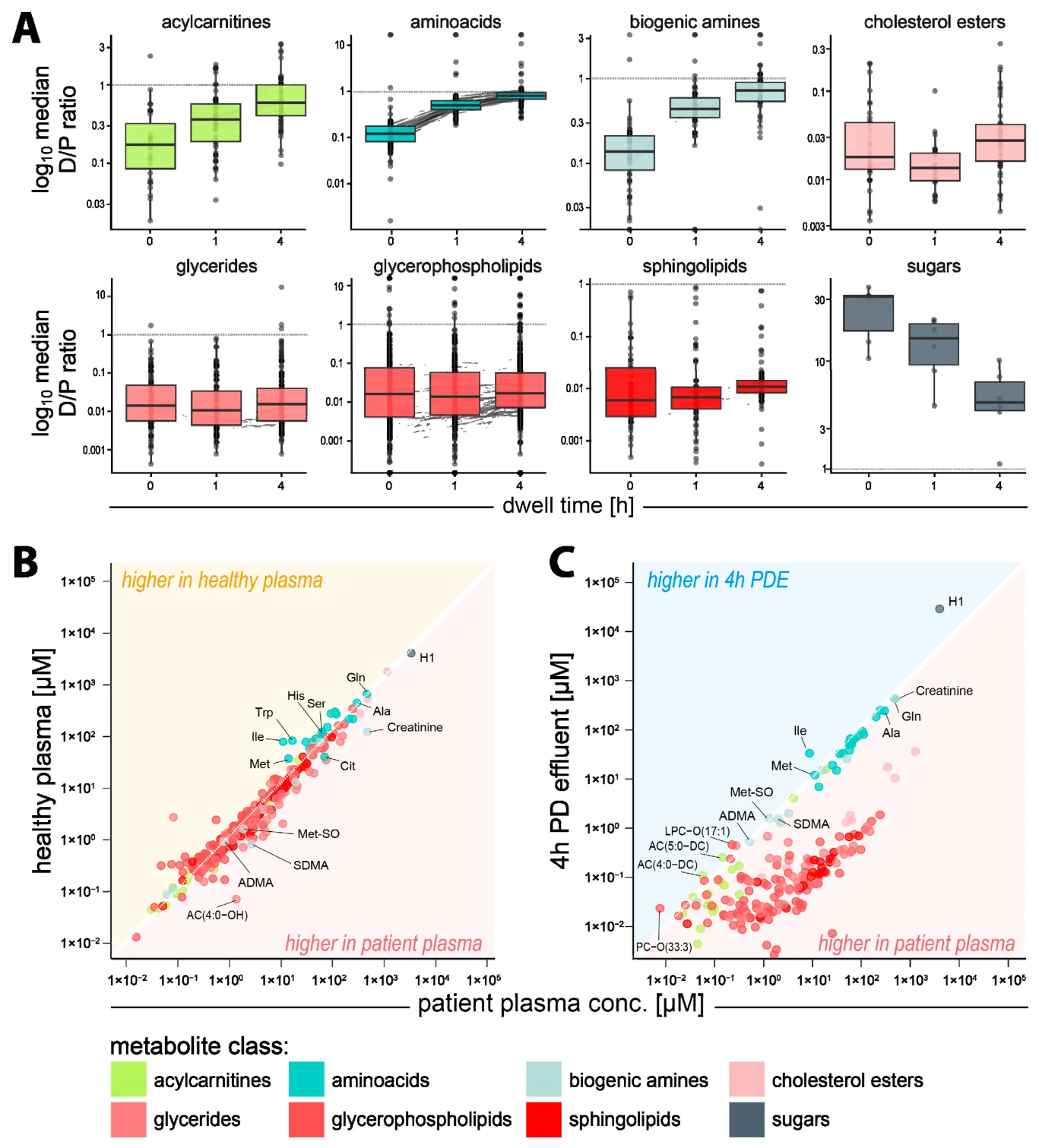
Metabolite class-specific transport during peritoneal equilibration. (**A**) Dialysate-to-plasma (D/P) ratios of individual metabolite classes at 0, 1, and 4 h of a standardized PET relative to matched plasma collected at the 2-h time point, illustrating class-specific equilibration kinetics. (**B**) Comparison of the plasma metabolome of PD patients and healthy controls. Only metabolites detected in both groups are shown. Selected metabolites with the largest differences between groups, including established uremic retention markers, are annotated. Symmetric dimethylarginine (SDMA), Asymmetric dimethylarginine (ADMA), amino acids are labeled in 3-letter codes. (**C**) Comparison of the 4-h PDE metabolome with matched PD patient plasma. Metabolites above the identity line are enriched in PDE, whereas metabolites below the identity line remain enriched in plasma after the 4-h dwell.

To quantify the transport efficiency of individual metabolite classes during clinically relevant PD dwell times, dialysate-to-plasma (D/P) ratios were determined throughout the PET. AAs and BAs exhibited the highest transport efficiency, with most metabolites approaching plasma equilibrium after 4 h and several exceeding plasma concentrations (D/P > 1) (Figure 2A). In contrast, glycerides, glycerophospholipids, sphingolipids, and cholesterol esters remained far from equilibrium (~D/P < 0.1), whereas acylcarnitines displayed highly species-dependent transport characteristics also during a PET. As expected, glucose concentrations declined continuously throughout the dwell, reflecting systemic absorption of the osmotic agent.

Comparison of PD patient plasma with healthy controls revealed reduced circulating AA concentrations together with modest increases in lipid species and elevated uremic retention solutes, including creatinine (q = 3.32 × 10^−6^), citrulline (q = 0.0018), and symmetric dimethylarginine (SDMA) (q = 0.067) (Figure 2B, Table S2). Consistent with the observed class-specific transport kinetics, comparison of the 4 h PDE metabolome with matched plasma showed that 11 metabolites exceeded plasma concentrations, whereas most lipids remained plasma-enriched despite prolonged equilibration (Figure 2C and Figure S4). Conversely, several acylcarnitines and lipid species were detected only in PDE, suggesting differential transport and/or local generation within the peritoneal cavity. Together these findings indicate that the temporal remodeling of the PDE metabolome is governed not only by dwell time but also by metabolite class-specific transport properties.

### 3.3. The Prolonged Dwell Remodels the PDE Proteome Toward a Tissue Remodeling Phenotype

**Figure 3:**
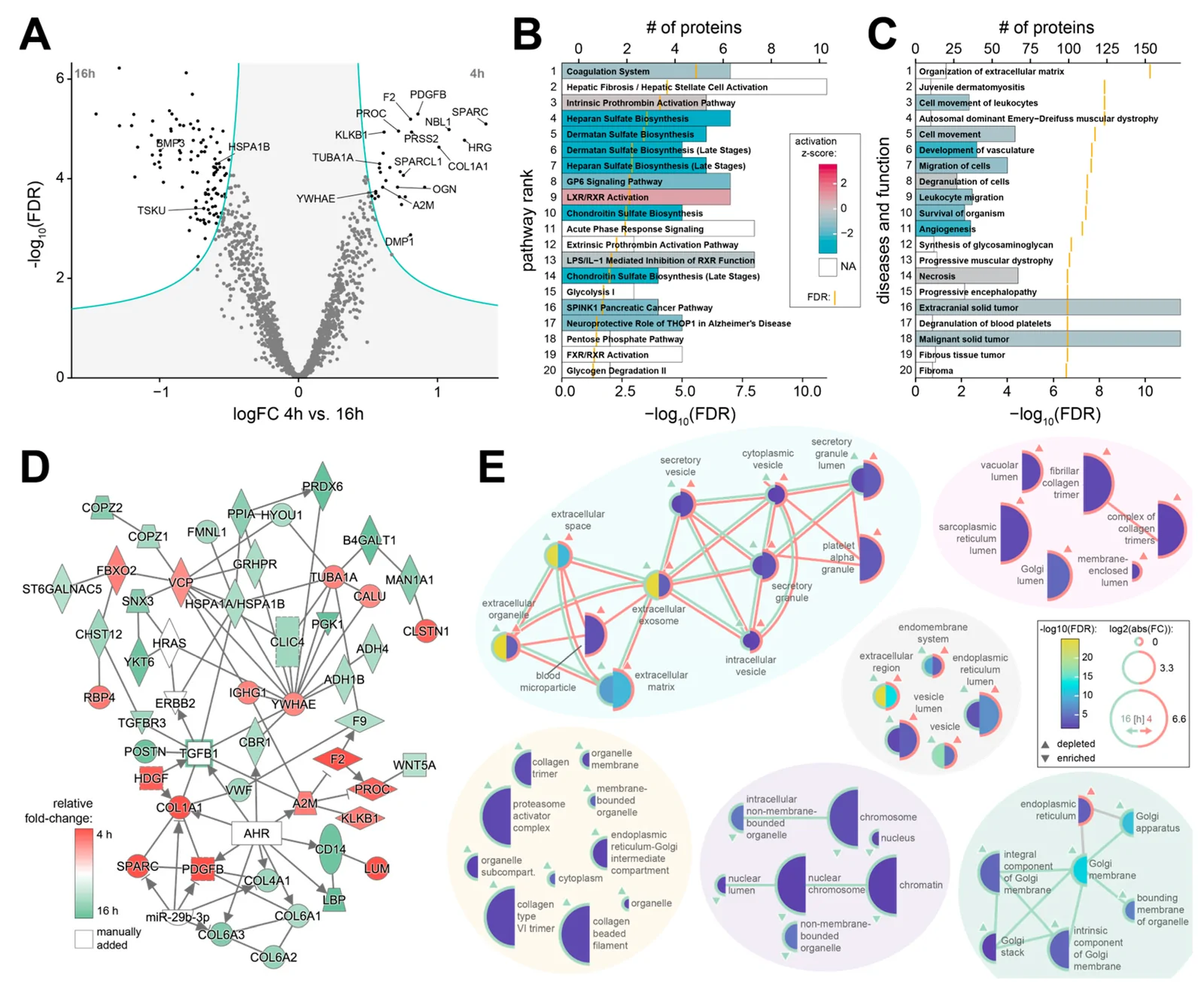
Prolonged dwell time influences the peritoneal dialysis effluent proteome. (**A**) Volcano plot of proteins differing between 4-h and overnight (16-h) dwell samples. A total of 133 proteins were significantly altered (FDR < 0.05), with 105 proteins enriched after the 16-h dwell and 28 proteins enriched after the 4-h dwell. Selected significantly regulated proteins are annotated. (**B**) Pathway Analysis of significantly regulated proteins. The top 20 enriched canonical pathways are ranked by significance (−log10 FDR). Bar color indicates the predicted activation z-score where available. (**C**) Diseases and Functions analysis (IPA) of significantly regulated proteins. The top 20 enriched biological functions and disease annotations are shown ranked by significance (−log10 FDR). (**D**) Protein interaction network of significantly regulated proteins together with selected upstream regulators identified. Only connected nodes are displayed. Proteins are colored according to their relative abundance after the 4-h (red) or 16-h (green) dwell. Selected upstream regulators and the PD-associated microRNA miR-29b-3p were added manually. (**E**) Gene Ontology Cellular Component (GO-CC) enrichment analysis of proteins enriched after the 4-h and 16-h dwells. Pie charts indicate enrichment of proteins after the 4-h (red) or 16-h (green) dwell, while node size and color represent enrichment significance (−log10 FDR) and fold enrichment, respectively.

Multiplexed quantitative proteomics of PDE collected after 4 h and overnight (16 h) dwells identified 2970 proteins, of which 133 changed significantly with dwell time (q < 0.05) (Figure 3A, Table S3). The majority (105 proteins, 79%) accumulated during the overnight dwell, whereas only 28 proteins were enriched after 4h, demonstrating progressive temporal remodeling of the PDE proteome.

Functional enrichment analysis revealed that dwell time-dependent proteins were predominantly associated with coagulation, extracellular matrix (ECM) remodeling, glucose metabolism, and hepatic fibrosis/hepatic stellate cell activation (Figure 3B). Analysis of enriched diseases and functions highlighted cell migration, angiogenesis, vascular development, and ECM organization among the top enriched terms (Figure 3C), suggesting coordinated changes in proteins involved in tissue remodeling and cellular motility throughout the dwell. Upstream regulator analysis yielded established mediators of peritoneal remodeling, including transforming growth factor β1 (TGFB1), tumor necrosis factor α (TNFA), and SNAI1, while network analysis integrating significantly regulated proteins with predicted upstream regulators identified YWHAE (a signal transducer), aryl-hydrocarbon receptor (AHR), and valosin-containing protein (VCP, p97) (involved in protein quality control) as central interaction hubs (Figure 3D; Tables S4 and S5). AHR showed high network connectivity, but its activation z-score (−0.038) did not support a defined direction of activity. Modeling of metabolite–protein associations further identified relationships between metabolites and AHR-associated proteins, without defining the direction of AHR activity (Figure S5). Cellular component analysis further demonstrated a shift in the molecular composition of the PDE proteome with increasing dwell time (Figure 3E). While proteins identified after both dwell times shared extracellular and vesicle-associated localizations, proteins accumulating during the overnight dwell were additionally enriching for Golgi-associated processes and reduced in nuclear and chromatin-associated components, consistent with altered cellular contributions to the PDE proteome over time.

### 3.4. Multi-Omics Integration Resolves the Local and Systemic Origin of the PDE Proteome

**Figure 4:**
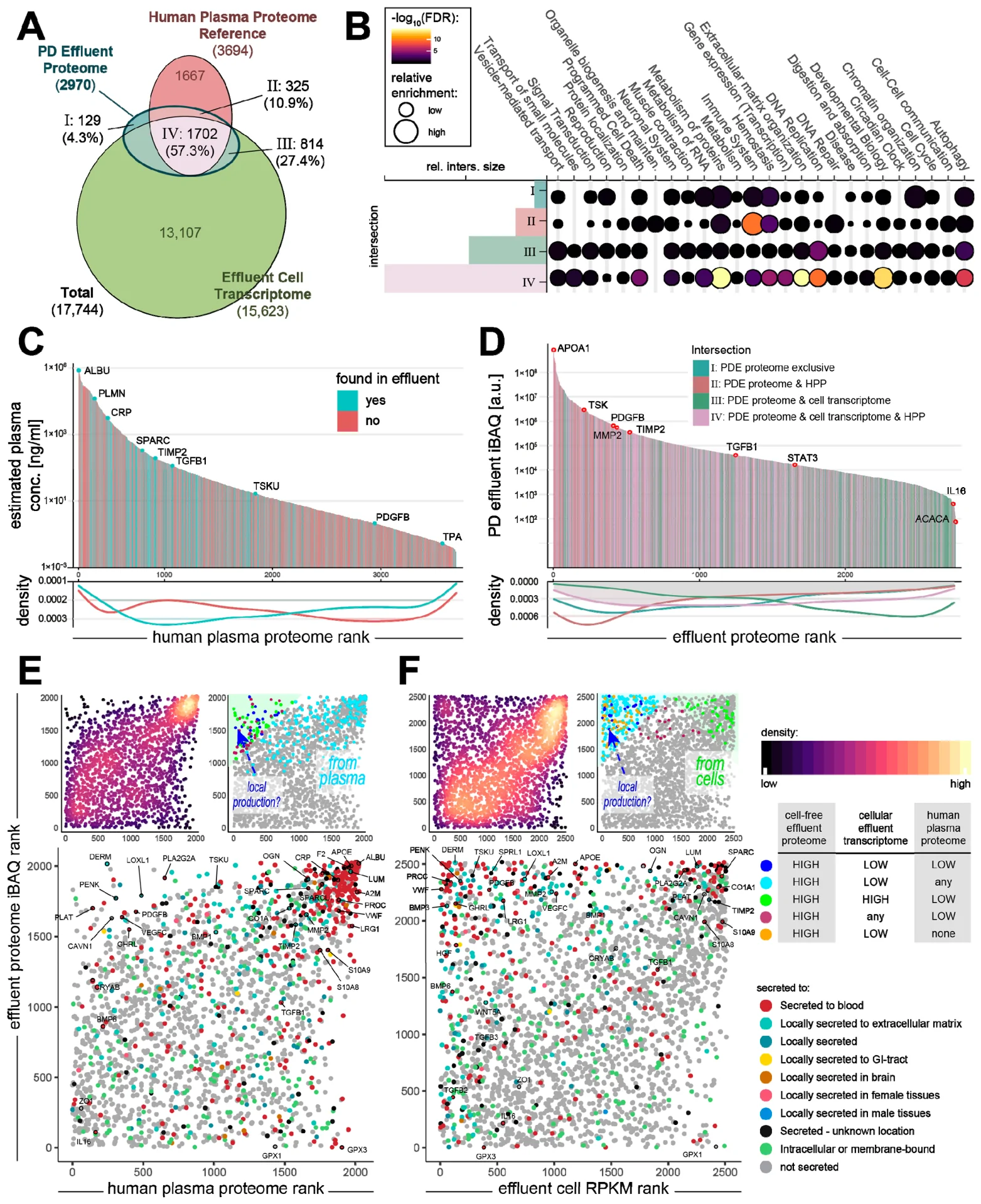
Integrative multi-omics reveals the putative molecular source of proteins detected in peritoneal dialysis effluent (PDE). (**A**) Qualitative overlap between proteins identified in cell-free PDE, transcripts detected in effluent cells, and the Human Plasma Proteome (HPP) reference dataset. Percentages indicate the proportion of proteins assigned to each intersection relative to all proteins detected in PDE. (**B**) Reactome enrichment analysis of proteins assigned to the four intersections shown in (**A**), demonstrating distinct biological functions associated with plasma-associated proteins, effluent cell-associated proteins, mixed-origin proteins, and proteins uniquely detected in PDE. (**C**) Distribution of proteins detected in PDE across the ranked Human Plasma Proteome. The upper panel shows estimated plasma protein abundance, with proteins identified in PDE highlighted in blue. Lower panel: density distribution of proteins. (**D**) Ranked abundance of proteins detected in PDE. Proteins are colored according to the source attribution shown in (**A**). (**E**) Rank-based source attribution based on quantitative comparison of PDE protein abundance with the Human Plasma Proteome. The upper density plot compares the abundance distributions of proteins detected in PDE and plasma. The lower scatter plot identifies proteins that are overrepresented or underrepresented in PDE relative to plasma. Proteins enriched in PDE despite low plasma abundance are consistent with local production, whereas proteins following plasma abundance likely reflect filtration from the systemic circulation. The lower panel indicates the predicted secretory status of proteins according to Uhlén et al. [20]. (**F**) Rank-based source attribution based on quantitative comparison of PDE protein abundance and effluent-cell transcript abundance. Proteins showing high abundance despite low transcript levels are consistent with plasma-associated proteins, whereas proteins with concordantly high transcript and protein abundance support production by free-floating effluent cells. Proteins exhibiting high abundance in PDE despite low plasma abundance and limited transcript expression represent candidate proteins originating from resident peritoneal cells. The lower panel indicates predicted protein secretion as in (**E**).

To distinguish proteins potentially originating from the systemic circulation from those produced within the peritoneal cavity, the PDE proteome was integrated with the transcriptome of effluent cells and a reference human plasma proteome. This analysis enabled classification of proteins according to their overlap with plasma proteins and effluent-cell transcripts, into likely plasma-associated, cell-associated, mixed local/systemic, and PDE-exclusive categories, providing an indication of their putative source. Of the 2970 proteins identified, 1702 (57.3%) were detected in all three datasets and therefore classified as mixed local/systemic proteins, indicating contributions from both plasma and local cellular sources (Figure 4A). A further 325 proteins (10.9%) overlapped exclusively with the plasma proteome, consistent with predominantly systemic origin (“plasma-associated”), 814 (27.4%) were shared only with the effluent cell transcriptome therefore considered “cell-associated”, and 129 (4.3%) were PDE-specific. Functional enrichment supported these assignments: plasma-associated proteins were enriched in hemostatic and immune pathways, cell-associated proteins in DNA replication, autophagy, and intracellular metabolism, whereas mixed local/systemic proteins were enriched in ECM organization, developmental biology, protein metabolism, and DNA replication (Figure 4B).

Proteins detected in PDE covered a broad plasma concentration range, however, mapping onto the reference plasma proteome revealed a clear bias towards highly abundant plasma proteins (Figure 4C). Accordingly, plasma-associated proteins predominated among the most abundant PDE proteins, whereas cell-associated proteins were at the lower end of the abundance range and mixed local/systemic proteins spanned the full dynamic range. Rank-based comparison further supported these source assignments (Figure 4D). Rank-based correlations showed that highly abundant PDE proteins largely mirrored plasma abundance and carried blood-specific secretory annotations, including canonical plasma proteins such as albumin (ALB), C-reactive protein (CRP), and prothrombin (F2) (Figure 4E,F). In contrast, 37 proteins ranked high in PDE and transcript abundance in effluent cells, supporting a cellular origin, whereas 183 proteins were abundant in PDE but showed low effluent-cell expression, consistent with systemic filtration (Table S6). Notably, 23 proteins ranked high in PDE, had low expression in effluent cells and were without corresponding abundance in plasma, making them consistent with origin from resident peritoneal cells. The majority of those contained secretory signals towards the bloodstream and local secretion. Additional 11 ranked high in PDE (WISP2, CLC11, MLRV, SRCH, MLRS, H15, SFRP3, RS18, ANGPT2, ANF, and RGMC) were absent from the effluent-cell transcriptome altogether, identifying them as candidate markers of resident peritoneal tissue.

## 4. Discussion

PD effluent has traditionally been regarded primarily as a surrogate of peritoneal transport efficiency and, more recently, as a source of candidate biomarkers [24]. Our findings substantially extend this perspective by establishing the first time-resolved systems-level framework integrating molecular composition, transport dynamics, and molecular origin of the PD effluentome. This multidimensional approach demonstrates that the molecular composition of PD effluent is continuously shaped by selective transperitoneal transport, ongoing biological activity within the peritoneal cavity, and contributions from multiple systemic and local cellular sources. Rather than representing a passive ultrafiltrate of plasma, the PD effluentome emerges as a dynamic molecular ecosystem reflecting the integrated consequences of transport physiology and tissue biology. This distinction changes how PD effluent should be interpreted and provides a conceptual framework for future mechanistic studies of the peritoneal membrane and the rational development of effluent-derived biomarkers.

The temporal metabolomics analysis demonstrates that molecular transport across the peritoneal membrane is governed by physicochemical properties extending beyond molecular size alone. While the classical three-pore model successfully explains the movement of water and many small solutes across the peritoneal membrane, it assumes diffusion to be primarily determined by molecular radius and concentration gradients [9,10,11,25,26,27,28]. Our findings complement this concept by revealing pronounced class-specific transport behavior, with rapid equilibration of hydrophilic amino acids and biogenic amines, whereas lipids remained markedly underrepresented in effluent even after prolonged dwell times.

Acylcarnitines provide an informative model for understanding transperitoneal transport because they share a common chemical backbone while increasing chain length progressively increases hydrophobicity while reducing aqueous solubility. Consistent with this, short-chain acylcarnitines equilibrated rapidly, whereas medium- and long-chain species showed progressively delayed equilibration. These findings suggest that hydrophobicity is an important determinant of transperitoneal transport, extending previous models that primarily emphasized molecular size, charge, and protein binding as dominant predictors of dialysate-to-plasma equilibration [25]. Acylcarnitines are central to fatty acid metabolism and have been linked to insulin resistance, glucolipotoxicity, and metabolic dysfunction. Their association with adverse clinical outcomes in dialysis patients highlights the importance of understanding their transperitoneal transport and retention kinetics [29,30,31]. A small set of metabolites exhibited transient concentration peaks followed by subsequent declines during the dwell, suggesting contributions from local secretion, metabolism, or reabsorption. Together, these observations demonstrate that the PD effluentome cannot be interpreted as a simple diffusion filtrate but instead represents the integrated product of selective transport, local peritoneal biology, and metabolite-specific physicochemical properties.

Although prolonged dwell times resulted in the expected accumulation of many plasma-associated proteins, the proteins undergoing the greatest temporal regulation were predominantly associated with ECM organization and tissue remodeling and repair, vascular remodeling, angiogenesis, and coagulation. These coordinated changes indicate that PD effluent captures an evolving biological response of the peritoneal membrane rather than simply the progressive accumulation of filtered plasma proteins. Predicted upstream regulators including TGFB1, TNF, and SNAI1 converge on pathways central to fibrosis, inflammation, metabolic adaptation, and endothelial remodeling, processes that are well established drivers of long-term peritoneal membrane dysfunction [32,33,34]. AHR was not detected in PDE but was expressed by effluent cells and emerged as a highly connected node within the PD effluentome. Although its activation score did not support either activation or inhibition, associations between metabolites and AHR-related proteins point to AHR-associated signaling as a potential link between the metabolic and proteomic landscapes of the PD effluentome. Given the responsiveness of AHR to endogenous metabolites and gut-derived uremic toxins, together with its established involvement in inflammation, oxidative stress, and ECM remodeling, this association warrants further investigation in the context of peritoneal remodeling during PD [34,35,36,37,38]. Taken together, analysis of PD effluent could provide a dynamic molecular snapshot of ongoing tissue adaptation and injury occurring within the peritoneal cavity.

By integrating complementary molecular layers additional to proteomics of the PD effluent [18,39], it was for the first time possible to assign the most likely origin of proteins found in the PD effluent. With this multi-omics approach we show that the majority of effluent proteins originate from mixed systemic and local sources, while distinct subsets can be attributed predominantly to plasma, effluent cells, or resident peritoneal tissue. This source-attribution framework substantially improves the biological interpretation of effluent proteins by separating transport from local production and identifying candidate proteins most consistent with secretion by resident peritoneal cells. Importantly, this framework adds an additional biological dimension to biomarker research. Whereas biomarker discovery has traditionally focused on identifying molecules whose abundance changes with disease or treatment [24,40,41], our approach provides mechanistic context by determining why those molecules are present in effluent and whether they primarily reflect systemic physiology, membrane permeability, immune cell activity, or resident peritoneal tissue biology. Beyond PD, this integrative strategy establishes a general framework for studying complex biological fluids in which systemic filtration and local tissue-derived signals coexist, including bronchoalveolar lavage fluid, synovial fluid, and cerebrospinal fluid, where distinguishing circulating from locally produced molecules remains a major challenge for biomarker interpretation.

Despite its widespread use, clinical monitoring of PD remains largely limited to a small number of biomarkers and functional parameters, failing to capture the molecular complexity of solute exchange and peritoneal membrane biology [16,17,42]. Our findings demonstrate that candidate biomarkers, particularly in PD, should not be interpreted solely based on changes in abundance without considering the mechanisms underlying their appearance [43]. Proteins derived predominantly from plasma primarily reflect alterations in systemic concentrations or membrane permeability, whereas locally produced proteins are more likely to report biological activity within the peritoneal cavity. Integrating quantitative abundance with molecular source attribution therefore provides an additional layer of biological context that may improve biomarker specificity and facilitate the identification of markers reflecting local or systemic inflammation, ECM remodeling, vascular adaptation, or fibrosis. More broadly, this framework provides a foundation for integrating longitudinal multi-omics with clinical phenotyping to better understand peritoneal membrane adaptation, identify molecular signatures preceding membrane failure, and support the development of precision medicine strategies in PD.

The relatively small study cohort (n = 6) limits the generalizability of the identified molecular signatures, which will require validation in larger, independent PD cohorts. The present study therefore provides a discovery and reference framework, while the identified molecular signatures require validation in larger, independent PD cohorts. A further limitation is that profiling with proteomics so far still required complete drainage of the peritoneal cavity to obtain sufficient effluent volumes, restricting protein analyses to the standard 4 h PET and overnight dwell and precluding the temporal resolution achieved for metabolomics. The source-attribution strategy relies on integration with bulk-transcriptomics and reference plasma datasets and therefore identifies putative molecular sources but not direct experimental validation of secretion by specific resident cell populations. In particular, detection in plasma or effluent cells does not establish exclusive derivation from either compartment. Future studies combining single-cell technologies and targeted protein and metabolite tracing approaches will be required to experimentally validate and further refine these assignments. Longitudinal investigations linking these molecular signatures to membrane function, structural remodeling, and long-term clinical outcomes will be required to determine their diagnostic and prognostic utility.

## 5. Conclusions

In conclusion, by defining the composition, transport dynamics, and molecular origin of the PD effluentome, this work establishes the first systems-level framework describing how PD effluent is generated. Rather than representing a passive dialysate, the PD effluentome emerges as a dynamic molecular ecosystem shaped by selective transport, continuous tissue biology, and multiple cellular and systemic sources. Integrating metabolomics, proteomics, and transcriptomics transforms PD effluent from a largely descriptive clinical sample into a biologically interpretable systems-level readout of peritoneal membrane health, providing a reference for future mechanistic studies, biomarker development, therapeutic monitoring, and ultimately precision medicine in PD.

## Data Availability

All mass spectrometry data have been deposited into the ProteomeXchange Consortium (http://proteomecentral.proteomexchange.org, accessed on 20 June 2026) via the PRIDE partner repository with dataset identifier PXD049292. All transcriptomics array data have been deposited into ArrayExpress with dataset identifier E-MTAB-5462.

## Supplementary Materials

The following supporting information can be downloaded at https://www.mdpi.com/article/10.3390/medsci14040496/s1, Figure S1: Metabolome profiles of PD effluent at all sampling time-points, and patient and healthy plasma; Figure S2: Significantly changing (ANOVA, Tukey’s post hoc) metabolites between sampling time points; Figure S3: Acylcarnitines: Relationship between molecular mass, solubility and 4 h Dialysate-to-Plasma ratio; Figure S4: Comparison of the PDE of different time points to plasma concentrations—dialysate-to-plasma (D/P) ratios; Figure S5: Possible regulatory effect of the aryl-hydrocarbon receptor (AHR); Table S1: Metabolomics; Table S2: Metabolomics healthy vs. PD patients plasma; Table S3: Proteomics IDs; Table S4: Upstream Regulators; Table S5: Highly Connected Features Network; Table S6: Multi Omics-Origin.

## Abbreviations

AAs	amino acids
ADMA	asymmetric dimethylarginine
AHR	aryl hydrocarbon receptor
BAs	biogenic amines
CHAPS	3-[(3-cholamidopropyl) dimethylammonio]-1-propanesulfonate
CPLL	combinatorial peptide ligand libraries
D/P	dialysate-to-plasma
ECM	extracellular matrix
EDTA	ethylenediaminetetraacetic acid
FASP	filter-aided sample preparation
FDR	false discovery rate
GO-CC	Gene Ontology Cellular Component
HCD	higher-energy collision-induced dissociation
HPLC	high performance liquid chromatography
HPPP	Human Plasma Proteome Project
iBAQ	intensity-based absolute quantification
IPA	Ingenuity Pathway Analysis
LCFA	long-chain fatty acids
LC-MS	liquid chromatography mass spectrometry
LOD	limit of detection
MCFA	medium-chain fatty acids
PD	Peritoneal dialysis
PDE	peritoneal dialysis effluent
PET	peritoneal equilibration test
RPKM	reads per kilobase per million mapped reads
SCFA	short-chain fatty acids
SDMA	symmetric dimethylarginine
TEAB	triethylammonium bicarbonate buffer
TGFB1	transforming growth factor β 1
TMT	tandem mass tag
TNFA	tumor necrosis factor α
VCP	valosin-containing protein
VLCFA	very long-chain fatty acids
