# Supplementary material for: The PD Effluentome—A Multi-Omics Atlas Defining the Composition, Transport Dynamics, and Molecular Origin of Peritoneal Dialysis Effluent
Source: Med Sci (Basel). 2026 Aug 19;14(4):496. doi: 10.3390/medsci14040496 (PMC13515947; doi:10.3390/medsci14040496)
Supplement: Supplementary file 1 [file medsci-14-00496-s001.zip › Supplementary Figures.pdf]

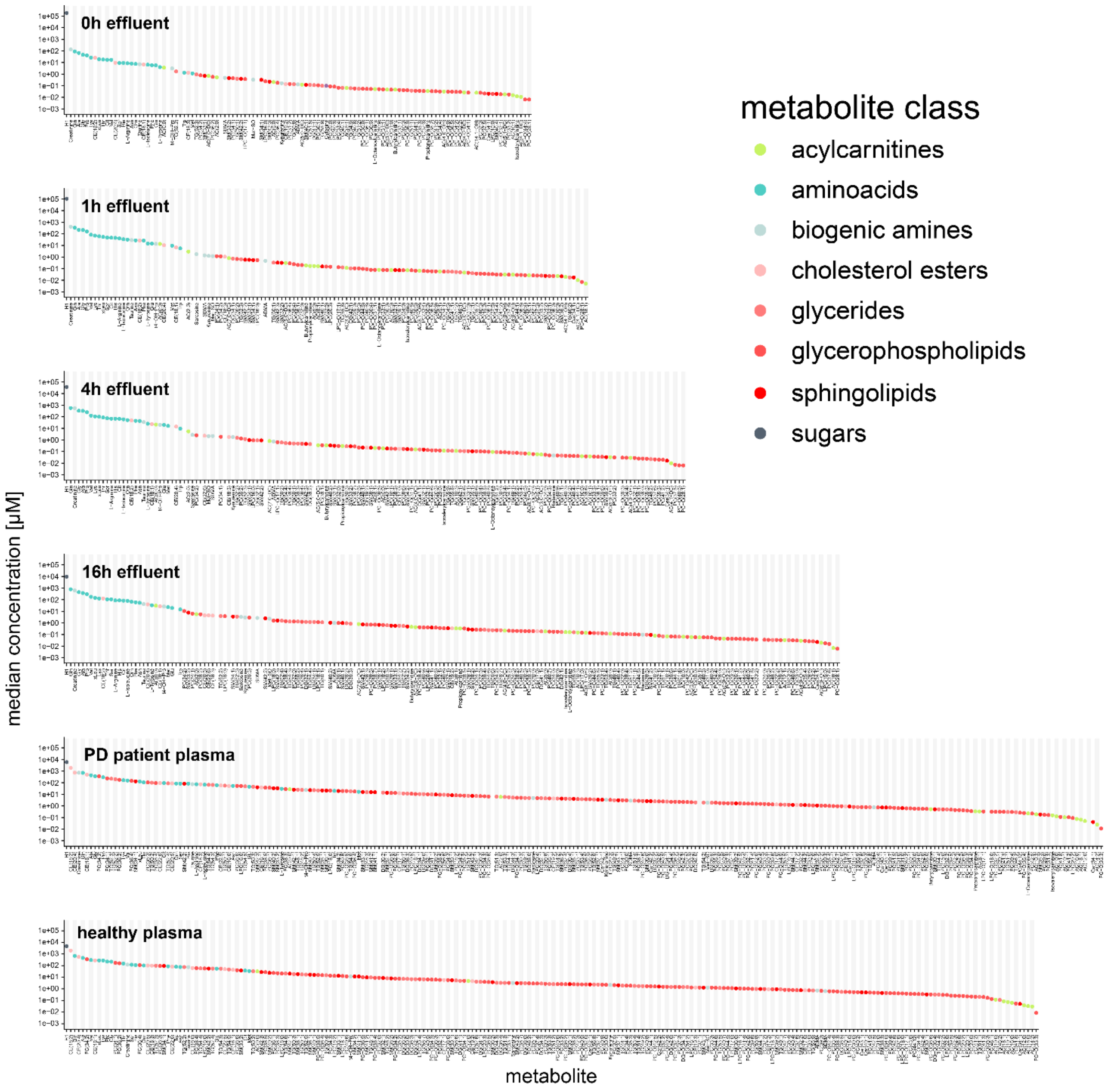

Figure S1: Metabolome profiles of PD effluent at all sampling time-points, and patient and healthy plasma. Metabolites are only shown, if they were detected in >50% patients.

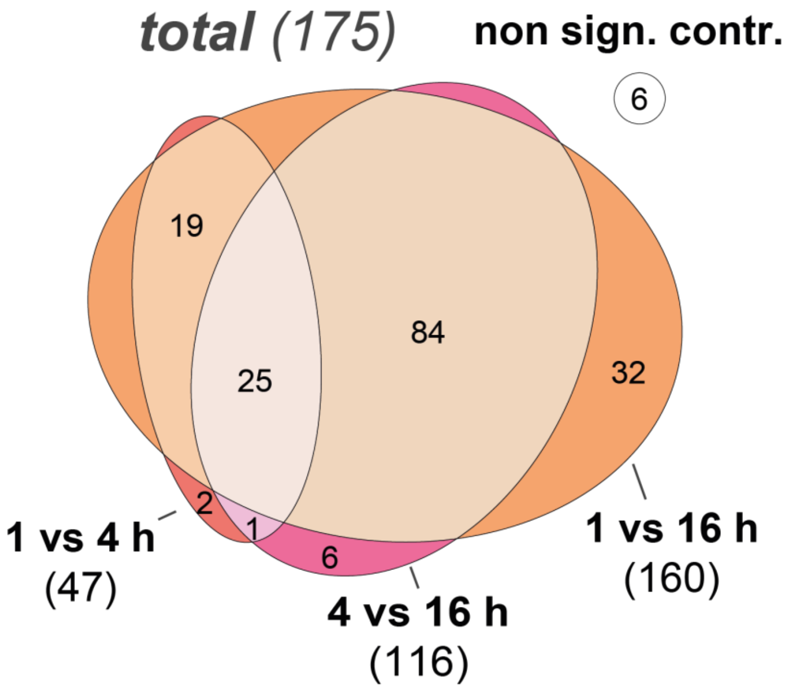

Figure S2: Significantly changing (ANOVA, Tukey's post hoc) metabolites between sampling time points.

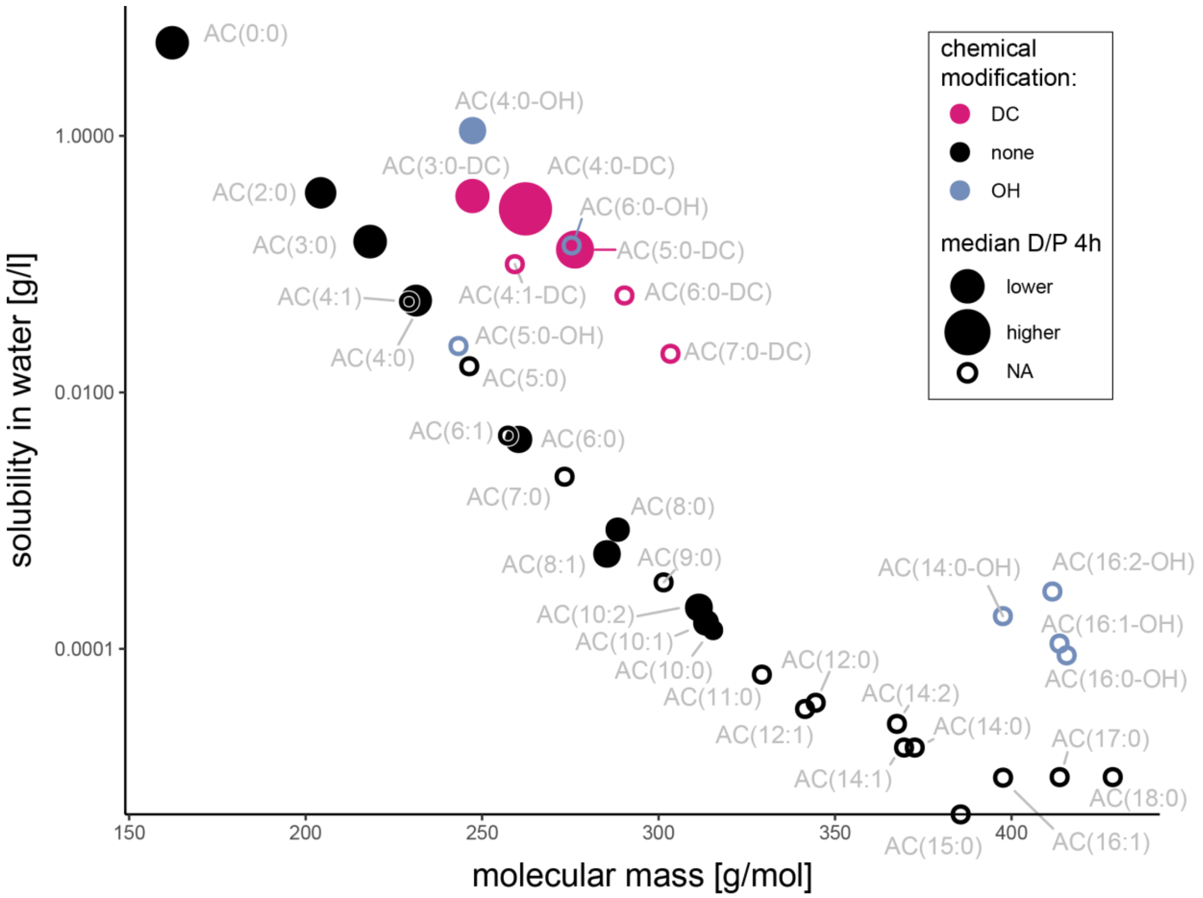

Figure S3: Acylcarnitines: Relationship between molecular mass, solubility and 4h Dialysate-to-Plasma ratio. Solubility of acylcarnitine species decreases with increasing molecular mass, which directly relates to the bound fatty acid chain length. The transport properties (Dialysate-to-Plasma ratio, D/P) at 4h of dwell of the individual acylcarnitines are indicated by bubble size. Some acylcarnitines were not detected in plasma or effluent material and D/P ratios could not be calculated.

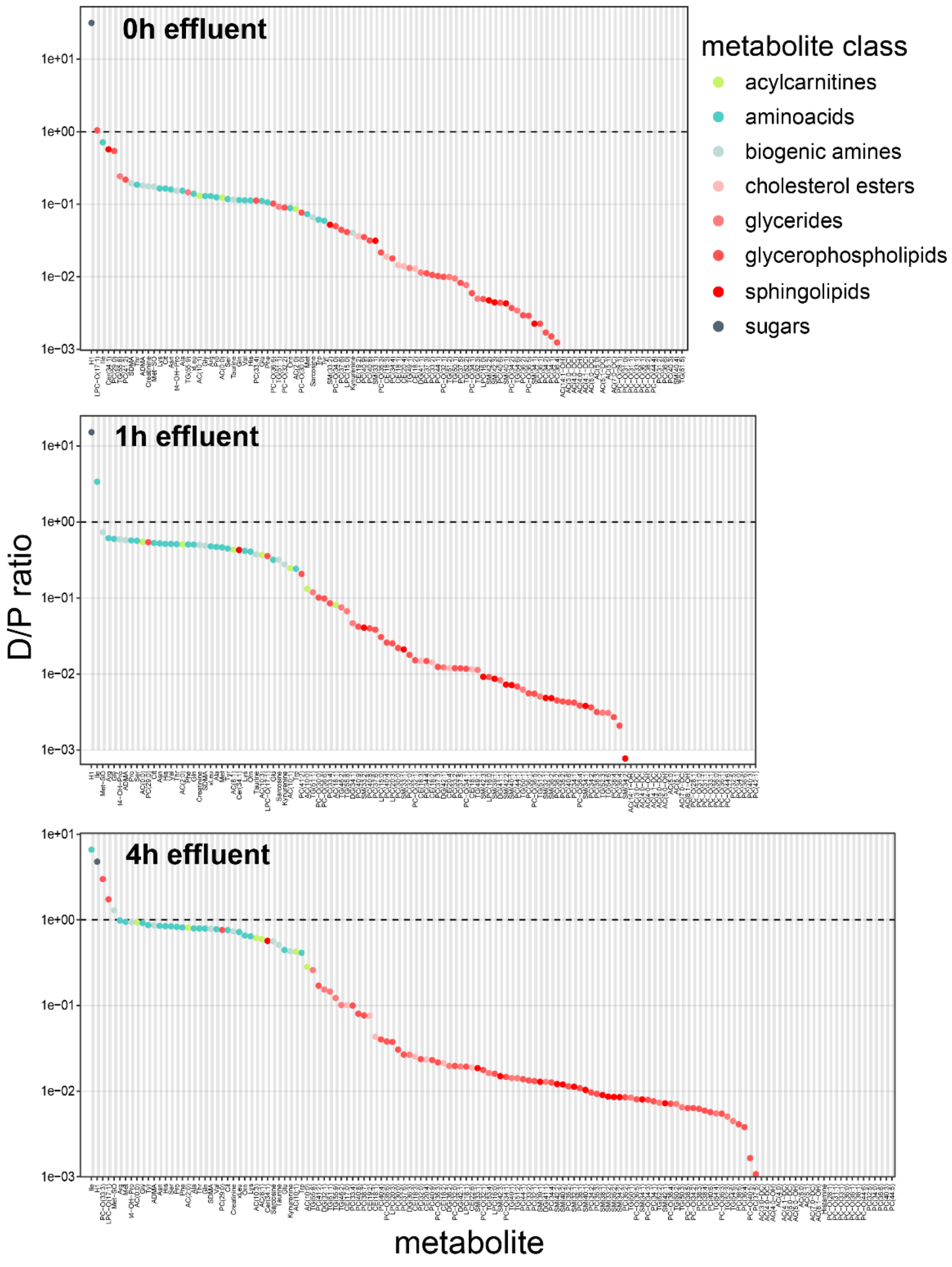

Figure S4: Comparison of the PDE of different time points to plasma concentrations – dialysate-to-plasma (D/P) ratios. Metabolome with matched PD patient plasma. Metabolites above the identity line are enriched in PDE, whereas metabolites below the identity line remain enriched in plasma

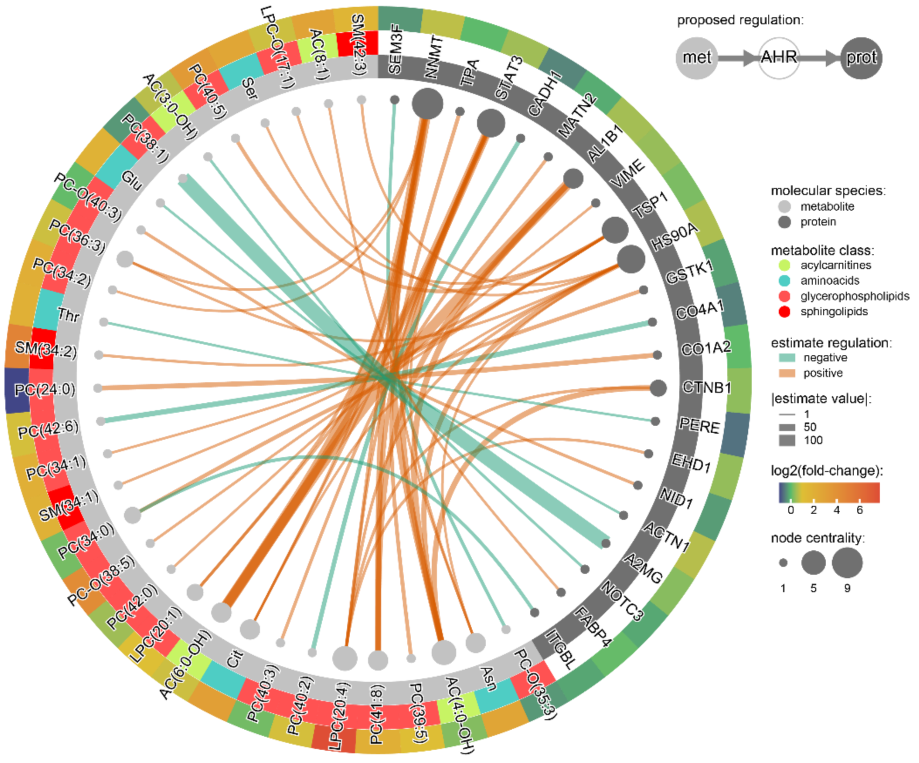

Figure S5: Possible regulatory effect of the aryl-hydrocarbon receptor (AHR). All metabolites were tested for their association with downstream elements of AHR and the top 50 interactions are shown. The estimated value indicates the effect size. $\log_2$(fold-change) shows the regulation between 4h and 16h. Node size reflects the amount of connected edges.
